# The Epidemiology of Burn and Lethal Area of Fifty Percentage (LA50) in Children in Shiraz, Southern Iran

**DOI:** 10.29252/wjps.10.1.66

**Published:** 2021-01

**Authors:** Seyedeh-Sara Hashemi, Mahdukht Mahmoodi, Hamid Reza Tohidinik, Ali Akbar Mohammadi, Davood Mehrabani

**Affiliations:** 1 Burn and Wound Healing Research Center, Shiraz University of Medical Science, Shiraz, Iran; 2 HIV/STI Surveillance Research Center, and WHO Collaborating Center for HIV Surveillance, Institute for Futures Studies in Health, Kerman University of Medical Sciences, Kerman, Iran; 3 Stem Cell Technology Research Center, Shiraz University of Medical Sciences, Shiraz, Iran

**Keywords:** Epidemiology, Burn, LA50, Children, Iran

## Abstract

**BACKGROUND:**

This study was conducted to assess the epidemiology of burn and lethal area of fifty percentage (LA50) in children in Shiraz, Southern Iran.

**METHODS:**

In this case series study, 619 hospitalized burn children from burn centers affiliated to Shiraz University of Medical Sciences, Shiraz, Iran from 2012 to 2016 were enrolled. Demographic characteristics of patients such as age, gender, place and cause of burn, and morality rate were evaluated. LA50 was measured using Probit analysis.

**RESULTS:**

The mean age of patients was 4.4±3.4 years. The mortality rate in burn patients was 8.7% and LA50 of total body surface area (TBSA%) ranged from 40.1% in 2012 to 68.3% in 2016. Although the number of male burn patients (65%) was more than females (35%), the mortality rate in females was more than males (11.4% vs. 7.2%). Scald and flame were the most common causes of burn.

**CONCLUSION:**

The findings in our burn center comparing burn patients to developed countries showed that LA50 and survival rate were lower denoting to an urgent necessity to promote current policies in burn care and prevention and to decrease the mortality rate too.

## INTRODUCTION

Accidents are still considered as the major non-communicable diseases in 21^st ^century affecting human activities, and among them, burn is considered as the most common one^1^. Fire accident is a type of burn occurring in both developed and developing countries leading to medical, psychological and economical losses for patients and their families in different age groups^2^^-^^4^. Factors such as life style, economic and cultural status of burn patients can affect the intensity of burn, and lead to mortality and morbidity, especially in developing countries^5^^-^^7^. Worldwide, 195000 of deaths were shown to happen annually due to burn injury. 

In the USA, burn is the 4^th^ cause of mortality necessitating medical attention for about 2.5 million patients each year. Annually, more than 100,000 of burn patients are hospitalized, while 40%-45% of them are usually children and 25% of them are children younger than 20 yr old. About 6000 of burn patients may die annually and permanent disability occurs in 50% of these patients^1^^, ^^8^^, ^^9^.

Children and teenagers are the most vulnerable groups facing the risk of accidents such as burn as the major cause of morbidity and mortality with serious social and economic subsequences ^10^^-^^12^. Most of burns occur in the first decade of life of children imposing psychological effects on the mental health^13^. The reported mortality rates in burn injuries denote to the importance of the quality of undertaken medical care. LA50 has been introduced as an index showing the burn percentage leading to death in 50% of patients^14^.

To provide preventive measures and required facilities for the treatment and rehabilitation of burn patients, epidemiological studies seem essential to estimate the mortality and morbidity rates. The epidemiology of burn in Tabriz, Iran during 2000-2010 indicated many aspects of burn, regarding age and gender, causes and mortality rate^15^. In 2009, LA50 was evaluated in Shahid Motahari Burn Hospital in Tehran, Iran during Oct 2004 to Sep 2005 showing the LA50 of 52.77%^16^. 

An epidemiological assessment during 2007 to 2010 in Rasht, north of Iran on burn patients hospitalized in Velayat Hospital included the total body surface area (TBSA) without reporting LA50 for those patients demonstrating a high prevalence of burn injuries in Iran that requires an increase in knowledge of the society as well as adhering to safety procedures both at home and workplace^17^. In Imam Khomeini Hospital in Kermanashah, Western Iran during 2011-2012; LA50 was reported 50.82% in hospitalized patients^18^. LA50 in burn injuries in Tehran, Iran, among 28690 burn patients from 3 months old to 93 yr old was shown to be 64.7%^19^. 

We aimed to determine the epidemiology and LA50 in burn children in Ghotbedin-Shirazi and Amiralmomenin burn hospitals in Shiraz, southern Iran.

## MATERIALS AND METHODS

From 2012 and 2016, in this case series study, epidemiologic and demographic data of pediatric burn patients younger than 14 yr old referring to Ghotbedin-Shirazi (2012-2014) and Amiralmomenin (2015-2016) burn hospitals affiliated to Shiraz University of Medical Sciences, Shiraz, southern Iran were enrolled. Quality of burn care is significantly higher in Amiralmomeni Hospital (2015-2016) due to more sophisticated equipment's more burn intensive care unit (BICU) beds. Trained surgeons, nurses and make a more isolation rooms. Demographic information included age, sex, type and cause of burn, place and date of accident, TBSA and mortality of children. Statistical analyses were performed using SPSS software (ver.16, Chicago, IL, USA) and Stata (ver. 11). 

To compare mortality rate between two genders, Chi square test was used. LA50 with 95% confidence interval (CI) was determined for all burn patients in consecutive years. A *P-*value less than 0.05 was considered statistically significant. 

Data were gathered in a retrospective approach and the principles of confidentiality were ensured according to the Helsinki declaration of bioethics. 

The study protocol was approved by the institutional Review Board and medical Ethics Committee of our institution (Ethical code: IR.SUMS.REC.1399.952).

## RESULTS

Overall, 619 burn children hospitalized in Ghotbedin-Shirazi and Amiralmomenin burn hospitals were included. Mean age of patients was 4.4±3.4 yr (range: 1-14 yr old), among them, 65% were male and 35% were female. The burn extent was 22.4±16.8% (range: 1%-100%). Majority of burn children (80%) were between one and six years old, while 55.3% lived in rural and 44.7% lived in urban regions. Hot liquid (46.8%) and fire (25.5%) were the most common causes for burn during the study period (others: 27.7%) and 78.3% of burn accidents happened at home. Winter was the season with the most number of hospitalizations due to burn (28%). The mortality rate was 8.7% that in female patients was more than male subjects (11.4% vs. 7.2%, *p*=0.08). LA50 ranged from 40.1 in 2012 to 68.3 in 2016 (Table 1).

## DISCUSSION

Burn can be associated with a significant rate of mortality and is still of major health importance. Nowadays in developed countries, severe burn injuries have been rare cases among children as the number and severity of such injuries have declined due to preventive campaigns, educational programs and public health interventions^20^. Although the mortality rate of burn injury decreased from 15000 deaths in 1970 to 4500 deaths in 1996, but LA5O has illustrated an increasing trend from 30% of 80%, and burn is still one of the most important factor of mortality in the world^4^. 

**Table 1:** LA50 in burn children in Shiraz, Iran during 2012-2016

	Lethal Area 50 for TBSA (%) (95% Confidence Interval)		
Year	Male n=403	Female n=216	Total n=619
2012, n=132	34.39 (20.1-76.5)	44.4 (26.2-100)	40.1 (34.3-100)
2013, n=138	49.6 (47.3-60.6)	42.8 (40.4-56.3)	47.6 (46.4-51.5)
2014, n=112	61.4 (55.3-85.1)	52.7 (50.8-65.4)	57.6 (54.1-66.3)
2015, n=113	64.1 (58.8-82.6)	62.7 (59.6-100)	62.27 (58.6-75.4)
2016, n=124	67.1 (40.0-79.0)	71.2 (61.7-100)	68.3 (60.9-66.61)

In the United States, children and young adults are the most vulnerable groups facing burn injuries, while hot liquids and flame are the most common causes of burn among children and young adults. A similar finding was already confirmed in Iran^4^. In our study, the mortality rate among children suffering from burn injuries was 8.9%, while males were affected more than female population. LA50 increased from 40.1% in 2012 to 68.3% in 2016, statistic shows that half of patients were with 30.1% TBSA, while the rate later increased to 68.3%. In China, the LA50 of 94% was demonstrated for full thickness burns in the country^21^. 

In Taiwan, LA50 was reported to be 80%^22^, and 60% in the UK^23^. Most of patients were male (male: female ratio of 1.7:1) and in 2-4 yr age range group. The majority of injuries were noted in male patients under the age of 5 yr old, because this age group is considered more curious and is may not realize the danger of several agents as the causes of burn injuries. The most affected body region was trunk and hot liquid was responsible for most of burn injuries among 1256 patients (73.2%) ^24^. 

The findings in our burn center when compared with developed countries showed that LA50 and survival of burn patients were less denoting to an urgent necessity for prompt attention to promote current policies in public health issue and to decrease the mortality rate^25^. In Baghdad, Iraq, burn injury is still a prevalent health issue with an increasing trend imposing a household financial load, high mortality rate and disability occurring after burn injury mandating the need for planning burn prevention and care program^26^. 

In our study, the LA50 was much lower than developed countries^27^, so the quality of medical care may be undertaken less, while female gender, age, inhalational injury and extension of burn were independent risk factors of mortality in our burn patients. The high LA50 index was shown to be a high priority in Northern Iran revealing the need to enhance the public knowledge and the quality of the care for women, children, elderly who can be at risk of extensive burns^28^. LA50 was higher in children than adults, and the most significant predictors of mortality in burn patients were burn size, female gender and age^19^. 

Suicide has been reported as the most prevalent cause of mortality, especially among women of Iran^19^. Accomplished studies in Iran revealed a lower LA50% in comparison to developed countries. In Kermanshah Province, Iran, LA50 of 50.8% was for all patients, but a separate age group of children was not reported^16^. In Tehran, the annual LA50 was 52% in burn patients, and thermal burn was the most lethal one among burn causes with an approximately LA50 of 52%^16^.

Another study in Iran University of Medical Sciences in Tehran, Iran for LA50 in burn injuries of 28690 burn patients revealed LA50 of 64.7% for all patients, 62.3% for adults and 72% for children^19^. Children were more resistant to trauma of equal-size burns. They could also survive in higher rate for the same percentage of TBSA supporting the idea that LA50 decreased with age^23^^, ^^29^. 

Based on our findings, the type of burn was different in various body areas. We studied the LA50 during years 2012-2016 to determine the facilities and treatment methods as children have special physiological and anatomical conditions and are more vulnerable to sepsis and infection. High infection control, high quality facilities and using modern surgical methods of treatment seem to be effective factors to improve LA50^30^. In our study, there was an increase in LA50 rate in hospitalized children. An improvement in LA50 index can indicate the success in nursing management of burn patients and new treatment measures for pediatric burn patients including ICU care, early excision, grafting and so on. Early excision and skin grafting in burn wounds can decrease the hospital stay and septic complications among hospitalized patients. It is one of the effective factors widely used from 2016 in our hospitals by surgical teams and seems to be effective in improvement of LA50 in this center^30^. 

## CONCLUSION

LA50 in our center was shown to be lower than developed countries that may be due to financial problems and inadequate supports in developing countries. The difference in data between Ghotbedin-Shirazi and Amiralmomeni Burn hospitals may be due to presence of less facilities specialists and BICU beds in Ghotbedin-Shirazi Hospital in comparison to the Amiralmomenin Hospital. Our findings when compared with developed countries revealed that LA50 and survival of burn patients were lower demonstrating an urgent necessity for prompt attention to promote current policies and planning in public health issue and care of burn patients to decrease the mortality.
